# Measurement and interpretation of the Harare HIV combination prevention cascade in priority populations: a population survey of adolescent girls and young women and young men in Zimbabwe

**DOI:** 10.1136/bmjph-2025-002860

**Published:** 2025-08-28

**Authors:** Louisa Moorhouse, Jeffrey W Imai-Eaton, Tawanda Dadirai, Rufurwokuda Maswera, Tafadzwa Museka, Phyllis Mandizvidza, Freedom Dzamatira, Blessing Tsenesa, Timothy B Hallett, Constance Nyamukapa, Simon Gregson

**Affiliations:** 1 School of Public Health, Imperial College London, London, UK; 2 MRC Centre for Global Infectious Disease Analysis, School of Public Health, Imperial College London, London, UK; 3 Centre for Communicable Disease Dynamics, Department of Epidemiology, Harvard T.H. Chan School of Public Health, Boston, Massachusetts, USA; 4 Biomedical Research and Training Institute, Harare, Zimbabwe

**Keywords:** HIV, Zimbabwe, adolescent, primary prevention

## Abstract

**Introduction:**

HIV-negative adolescent girls and young women (AGYW), and men (ABYM), have disproportionately high HIV incidence in many African countries. We used a new HIV Prevention Cascade (HPC) approach to quantify levels of, and barriers to, prevention method use to guide interventions to increase effective uptake of primary HIV prevention.

**Methods:**

Data from the Manicaland HPC pilot study (2018–19; n=9803) in Zimbabwe were used to measure levels of sexual risk behaviour and construct HPCs for male condom, pre-exposure prophylaxis (females), voluntary medical male circumcision (males) and combination prevention use by HIV-negative sexually active AGYW (15–24 years) and male partners (15–29 years).

**Results:**

19% of AGYW (n=1140) and 37% of ABYM (n=955) who had started sex reported one or more HIV risk behaviour and met the definition of the priority populations for HIV prevention. Of these, 63% of AGYW and 87% of ABYM were motivated to use an HIV prevention method, 28% and 63% had access to a method and 16% and 53% used a method. Male condoms were the most commonly used prevention method, accounting for 97% of use in AGYW and 55% in ABYM. Barriers to motivation, access and capacity to use were reported for all priority populations and methods. Some barriers were common across HPCs (eg, lack of risk perception, social unacceptability and lack of acceptable provision); others were specific to particular prevention methods or priority populations (eg, lack of availability).

**Conclusion:**

HIV risk behaviours were commonly reported, but gaps in use of prevention methods exist among young people reporting these HIV risk behaviours in Manicaland. Population survey measurements of HPCs revealed large gaps in all steps in the cascade (lack of motivation, lack of access and lack of capacity to use prevention) and provided information on the reasons for these gaps that can aid in designing interventions that reduce new infections.

**WHAT IS ALREADY KNOWN ON THIS TOPIC**

**WHAT THIS STUDY ADDS**

- The study revealed notable gaps in motivation to use, access to and use of HIV prevention methods in both adolescent girls and young women and their potential male partners in east Zimbabwe.
- The measurements of barriers contributing to each gap in the HPCs pinpointed that interventions increasing motivation, access and capacity to effectively use prevention are needed to increase use of HIV prevention methods and demonstrated the importance of considering multilevel targets addressing this range of barriers for these interventions.

**HOW THIS STUDY MIGHT AFFECT RESEARCH, PRACTICE OR POLICY**

- This paper provides a new approach to driving the increases in primary prevention use needed to accelerate declines in new HIV infections.
- It does so by applying the Harare HIV-CPC framework to population surveys, using the data to identify which of the multiple possible barriers to prevention use are locally relevant, and determining the targeted interventions that need to be resourced and implemented.

## Introduction

 HIV incidence has declined in eastern and southern Africa due to protective changes in sexual behaviours^1 2^ and widespread availability of antiretroviral therapy (ART) reducing HIV infectiousness.^3 4^ However, reductions in incidence have been slower than targeted and the UNAIDS milestone of reducing global new infections to fewer than 500 000 per year by 2020 was missed.^5^,^7^ Incidence declines have varied between population subgroups across eastern and southern Africa.^8 9^ This heterogeneity has been attributed to a combination of variation in the provision of HIV prevention programmes and uptake of preventive behaviour,^10^ emphasising the need to improve understanding of why people are not adopting HIV prevention and for novel approaches to improve the use and impact of prevention methods. HIV-negative adolescent girls and young women (AGYW), in particular, experience high HIV incidence in the region and therefore have been identified as a priority population for targeting prevention.^11 12^

Despite the availability of efficacious HIV prevention methods, levels of use vary considerably by type of sexual relationship and between populations at risk of acquiring HIV. When used correctly, condoms reduce HIV transmission by 90%–95%.^13 14^ Voluntary medical male circumcision (VMMC) reduces HIV acquisition in men by 60%.^15^ Oral pre-exposure prophylaxis (PrEP) reduces acquisition risk by up to 90%, with good adherence.^16^ However, UNAIDS estimates a gap of 3 billion condoms a year in eastern and southern Africa.^6^ While VMMC expanded rapidly in the second half of the 2010s, the number of VMMC procedures reduced by half from 2019 to 2020 with service disruptions following the COVID-19 pandemic.^17^ PrEP use is a key tool in the 2025 UNAIDS roadmap for HIV prevention but gaps remain in the provision and demand for PrEP.^17^ PrEP targets aim to make PrEP available to all people at elevated risk of HIV infection—estimated to be 10 million people.^17^

Understanding multilevel components contributing to insufficient use of prevention methods, including demand, supply and structural barriers, is crucial to reaching global targets for HIV incidence reduction. The Harare HIV prevention cascade (HIV-HPC) has been proposed as a generic framework to be applied to multiple populations and both individual and combination (referred to as the Harare HIV combination prevention cascade or HIV-CPC) prevention methods,^18^ and has been developed through a series of consultations and literature review.^19^ It focuses on the identification of a priority population in need of HIV prevention methods, followed by three core steps, represented as sequential bars in a cascade: motivation to use, access to and effective use of the prevention method. Large gaps between successive steps in the cascade can be interrogated according to the frequencies of potential barriers that underpin most common reasons for these gaps. The sub-bars hypothesised in the HPC,^18^ identified from literature review, social-cognitive models and behavioural and epidemiological theories,^20^ represent these barriers and can suggest targets for interventions most likely to be effective at increasing use of the prevention method. One significant advantage of the HIV-HPC framework is that it can account for the contribution of multilevel barriers, including those operating at individual, social and structural levels, within one framework.

In this study, we carried out a pilot study in east Zimbabwe to measure and interpret the Harare HIV-CPC using data from a general population survey. We previously established that this is feasible and provides valid data on the gaps and barriers to use of prevention methods.^21^ In this study, we demonstrate the utility of collecting and interpreting the Harare HIV-CPC in a population survey using the examples of priority populations of AGYW and ABYM as potential male partners. In so doing, we provide valuable new insights into the types of interventions needed to increase use of prevention methods and to reduce new infections in these vulnerable groups.

## Methods

### Study setting and data source

Data were collected as part of the Manicaland HPC Study, conducted across eight study sites in east Zimbabwe (July 2018–December 2019), which represent urban, peri-urban, farming estates and subsistence farming areas. In each study site, a complete household census and questionnaire was conducted and household members, aged 15 years and above, were invited to participate in individual interviews. Data on sociodemographic characteristics, HIV knowledge, risk and prevention method use were collected using a questionnaire designed specifically to populate HPCs. Sensitive questions were asked using a secret voting method, whereby participants are not required to disclose their response to the interviewer and instead directly input their response to the data capture form.^22^ Data were collected via electronic data capture forms using Open Data Kit. Consent to complete the household questionnaire was collected from the head of the household (the questionnaire respondent). Consent to participate in the individual questionnaire, and, separately, to collect dried blood spots, was collected from all participants prior to the respective study activity. If participants were aged under 18 years, individual assent and parental consent was collected.

All participants completing the individual questionnaire were offered HIV counselling and testing (HTC) and were requested to provide a dried blood spot (DBS) sample for laboratory testing. HTC was conducted using the Zimbabwe Ministry of Health’s rapid testing algorithm and guidelines, based on the 2015 WHO recommendations.^23^ Where HTC was accepted, the HTC result was used as the final HIV status result. Where HTC was declined (eg, due to fear of testing) but consent and DBS were provided for laboratory testing, the same testing algorithm was completed at the Biomedical Research and Training Institute laboratory using the DBS.

This manuscript adheres to the Strengthening the Reporting of Observational Studies in Epidemiology (STROBE) statement for observational studies. A completed STROBE checklist is provided in online supplemental file 1.

### Data analysis

Data were restricted to HIV-negative females aged 15–24 years (AGYW) and males aged 15–29 years (ABYM). Proportions and 95% CIs were calculated for sociodemographic characteristics. Socioeconomic status was calculated as a wealth score taking into account reported sellable and non-sellable household assets split into quintiles.^24^ Descriptive statistics for participants’ sexual risk behaviours were calculated for survey participants who self-reported having ever had sex.

Harare HPCs^18 19^ were populated separately for males and females. The AGYW priority population for the cascades was defined as HIV-negative women aged 15–24 years who self-reported at least one risk behaviour in the last 12 months. The ABYM priority population was defined as HIV-negative men aged 15–29 years self-reporting ≥1 sexual risk behaviour in the last 12 months. This age range for ABYM, as potential male partners, was based on the age range of partners most commonly reported by AGYW in previous surveys in the study areas. Risk behaviours (identified through previous analyses of Manicaland cohort data^25^) were having multiple partners in the last 12 months; concurrent partners at the time of interview; recent transactional sex in the last month with any of the last three partners and reporting ≥1 non-regular partner in the last 12 months.

To create the main bars in the HIV-HPCs for individual HIV prevention methods, proportions and 95% CIs of the priority populations reporting motivation, access and effective use were calculated separately for male condoms, female condoms, PrEP and VMMC. If an individual reported currently using male condoms, female condoms or PrEP as a method of preventing HIV, they were defined as effectively using the respective prevention method. Effective use of VMMC was defined as having taken up full medical male circumcision. Exact definitions and full methods for populating the cascade have been validated and this validation is published separately.^21^ Definitions of each step are detailed in online supplemental file 2. Individuals who reported effectively using a particular HIV prevention method were assumed to be motivated and have access to that method.

Full extended HIV-HPCs reflecting explanatory factor sub-bars were created for male condoms, female condoms, PrEP and VMMC (men only). The frequencies of each of the explanatory factors among individuals who reported gaps in the main HPCs for each prevention method were measured and shown as sub-bars in the expanded cascade diagrams. Logistic regression was used to assess associations between sociodemographic characteristics and prevention method use, and differences between the HIV-HPCs.

HIV-CPCs were created to assess motivation, access and use of at least one prevention method for males (VMMC, male condoms, female condoms) and for females (PrEP, male condoms, female condoms). Stacked bar HIV-CPCs were created: first, with levels of motivation, access and effective use as proportions of the priority population; and then also broken down into each prevention method as proportions of each bar (motivation, access, effective use). Proportions of the priority population reporting motivation, access and effective use of at least one prevention method (male condoms, female condoms, PrEP, VMMC) were calculated.

### Patient and public involvement

Members of the public from communities where the study was carried out were consulted on the survey, key questions measuring the steps of the HPC and perceptions about using prevention methods among young people. Members of the public were involved in the conduct and dissemination of the research. Members of the public were recruited from the study areas as community research assistants in order to carry out the household census. A youth advisory board (YAB), consisting of young people from the study areas, was established at the start of the study to provide advice on: (1) the scientific design and practical implementation of the study; (2) the appropriateness of the research and intervention methods and tools for use with young people in the study areas; (3) methods of recruitment and retention of young people into the study; (4) how the study is being received by young people and other groups in the study areas and suggestions as to how problems arising can best be addressed; and to advise and assist with dissemination of results from the study and with formulating appropriate policy recommendations based on these results. Village leaders and headmen from all study sites were consulted regarding plans for dissemination within their respective communities.

### Statistical analysis

Analyses were carried out using Stata MP V.17. Tableau was used for data visualisations.

## Results

### Study population and HIV prevalence

Seventy-eight per cent (9803/12 647) of all eligible individuals completed the individual questionnaire. An HIV result was established for 95% (9339/9803) of individuals completing the individual questionnaire. Forty-six per cent (4286/9339) were adolescent and young people (AYP)—women aged 15–24 years or men aged 15–29 years. HIV prevalence was estimated at 2.76% (95% CI 2.14 to 3.55) in ABYM and 3.12% (95% CI 2.46 to 3.94) in AGYW.

### Sociodemographic characteristics

Approximately half of AYP participants were aged 15–19 years, comprising 48% of men and 53% of women (table 1). More AGYW than ABYM resided in urban sites (23% vs 17%) and were currently married (43% vs 25%). Around 90% of both ABYM and AGYW reported secondary or higher education. More than 50% of individuals lived in households in the poorest or second poorest socioeconomic quintile.

**Table 1:** Sociodemographic characteristics and self-reported sexual risk behaviours of HIV-negative adolescent and young people

	Female	Male
	15–24 years (n=2081) % (95% CI)	15–29 years (n=2079) % (95% CI)
Sociodemographic characteristics		
5-year age group		
15–19 years	53.4 (51.3 to 55.6)	47.7 (45.3 to 49.6)
20–24 years	45.6 (44.4 to 48.7)	31.6 (29.6 to 33.6)
25–29 years	–	21.0 (19.3 to 22.8)
Site type		
Urban	23.1 (21.4 to 25.0)	17.4 (15.8 to 19.1)
Peri-urban	26.4 (24.6 to 28.4)	22.0 (20.3 to 23.8)
Estates	21.6 (19.9 to 23.5)	23.0 (28.0 to 32.0)
Rural	28.8 (26.9 to 30.8)	30.7 (28.7 to 32.7)
Education		
None/Primary	11.3 (10.1 to 12.8)	8.9 (7.7 to 10.2)
Secondary/Higher	88.7 (87.2 to 89.0)	91.2 (89.9 to 92.3)
Marital status		
Never married	51.6 (19.5 to 53.8)	73.7 (71.8 to 75.6)
Currently married	42.9 (40.8 to 45.0)	24.8 (23.0 to 26.7)
Divorced/Separated	5.3 (4.4 to 6.3)	1.4 (1.0 to 2.0)
Widowed	0.2 (0.1 to 0.6)	0.1 (0.0 to 0.3)
Socioeconomic status		
Poorest	8.6 (7.4 to 9.9)	8.8 (7.7 to 10.1)
Second poorest	39.5 (37.4 to 41.6)	47.7 (45.6 to 49.9)
Third poorest	24.8 (23.0 to 26.8)	21.7 (20.0 to 23.5)
Fourth poorest	25.6 (23.8 to 27.5)	20.4 (18.7 to 22.1)
Least poor	1.5 (1.1 to 2.2)	1.4 (1.0 to 2.1)
Sexual risk behaviours		
Number reporting sexual debut*	1140	955
Had sexual debut	54.8 (52.6 to 56.9)	45.9 (43.8 to 48.1)
Age at first sex <18 years†	46.1 (43.3 to 49.1)	33.0 (30.1 to 36.0)
Had multiple partners in last 12 months†	4.6 (3.5 to 5.9)	21.5 (19.0 to 24.2)
Concurrent partners†	1.1 (0.7 to 2.0)	6.7 (5.3 to 8.5)
One or more non-regular partners in last 12 months†	13.3 (11.4 to 15.3)	31.8 (28.9 to 34.9)
Ever engaged in transactional sex†	8.7 (7.2 to 10.5)	14.7 (12.6 to 17.1)
Recent transactional sex in last month with any of last three partners†	8.0 (6.5 to 9.7)	7.2 (5.7 to 9.1)
Median age of last sexual partner†*	26.5	20
One or more of above risk behaviours‡†	18.5 (16.4 to 20.9)	37.1 (34.1 to 40.2)
Two or more of above risk behaviours‡†	4.2 (3.2 to 5.5)	10.6 (8.8 to 12.7)

* Reported as actual number not %*.*

† Restricted to those who have had sexual debut*.*

‡ Combining recent transactional sex, non-regular partners, multiple partners and concurrent partners*.*

### HIV risk behaviours

Fifty-five per cent of AGYW (15–24 years) and 46% of ABYM (15–29 years) had started sex (table 1). Among those who had started sex, 22% of ABYM reported multiple partners in the last 12 months compared with 5% for AGYW, and 32% of ABYM reported ≥1 non-regular partners in the last 12 months compared with 13% of AGYW. Few AGYW (1%) reported concurrent partnerships compared with ABYM (6%). Recent transactional sex was similar in AGYW and ABYM (8% vs 7%). The median age of the last partner reported by females was 26.5 years compared with 20.0 years reported by males. A markedly higher proportion of ABYM reported ≥1 risk behaviour compared with AGYW (37% vs 19%). A total of 37% (n=354) ABYM and 19% (n=211) AGYW met the definition for the prevention priority populations for HPCs.

### Associations of main bars with sociodemographic characteristics

Table 2 shows bivariate associations of sociodemographic characteristics with prevention use among the priority population at risk of HIV infection. Condom use was not significantly associated with age. Men aged 25–29 years had significantly lower odds of having VMMC compared with men aged 15–19 years (OR 0.39, 95% CI 0.18 to 0.83). ABYM who had completed secondary or higher education had 2.5 times the odds (95% CI 1.18 to 5.30) of using male condoms compared with those with no or primary education only. However, VMMC did not vary by education. Male condom use among AGYW did not vary by education level. Being currently married was associated with lower odds of male condom use compared with never being married in both men (OR 0.48, 95% CI 0.31 to 0.73) and women (OR 0.06, 95% CI 0.02 to 0.19). Prevention method use did not vary significantly by socioeconomic status.

**Table 2:** Unadjusted associations between sociodemographic characteristics and prevention method use in women and men in the priority population for HIV prevention cascades

	Male condoms				VMMC	
	Male (n=354)		Female (n=211)		Male (n=354)	
	OR (95% CI)	P value	OR (95% CI)	P value	OR (95% CI)	P value
5-year age group						
15–19 years	1.00		1.00		1.00	
20–24 years	0.86 (0.45 to 1.64)	0.637	0.96 (0.51 to 1.82)	0.912	0.63 (0.32 to 1.23)	0.177
25–29 years	1.04 (0.52 to 2.08)	0.901	–	–	0.39 (0.18 to 0.83)	0.014
Site type						
Urban	1.00		1.00		1.00	
Peri-urban	0.92 (0.41 to 1.81)	0.808	0.82 (0.34 to 2.01)	0.670	1.29 (0.58 to 2.86)	0.525
Estates	0.79 (0.43 to 1.46)	0.458	1.12 (0.48 to 2.62)	0.798	1.33 (0.65 to 2.74)	0.437
Rural	0.79 (0.41 to 1.54)	0.651	0.52 (0.22 to 1.24)	0.139	1.31 (0.58 to 2.95)	0.519
Education						
None/Primary	1.00		1.00		1.00	
Secondary/Higher	2.50 (1.18 to 5.30)	0.016	0.49 (0.22 to 1.10)	0.085	1.63 (0.60 to 4.38)	0.335
Marital status						
Never married	1.00		1.00		1.00	
Currently married	0.48 (0.31 to 0.73)	0.002	0.06 (0.02 to 0.19)	<0.001	0.59 (0.34 to 1.02)	0.059
Divorced/Separated	1.76 (0.56 to 5.49)	0.334	1.92 (0.87 to 4.24)	0.107	0.51 (0.14 to 1.80)	0.293
Widowed	1.00	–	1.37 (0.08 to 22.68)	0.825	1.00	–
Socioeconomic status						
Poorest	1.00		1.00		1.00	
Second poorest	0.67 (0.28 to 1.61)	0.369	0.65 (0.23 to 1.81)	0.405	0.96 (0.38 to 2.43)	0.930
Third poorest	0.62 (0.24 to 1.59)	0.321	0.83 (0.28 to 2.46)	0.730	0.58 (0.21 to 1.66)	0.312
Fourth poorest	1.23 (0.45 to 3.34)	0.687	0.87 (0.28 to 2.65)	0.802	0.69 (0.24 to 2.00)	0.495
Least poor	0.30 (0.04 to 2.13)	0.227	1.00	–	0.68 (0.06 to 7.16)	0.747

VMMC, voluntary medical male circumcision.

### HIV prevention cascades for AGYW in the priority population (reporting sexual risk behaviours)

**Figure 1:**
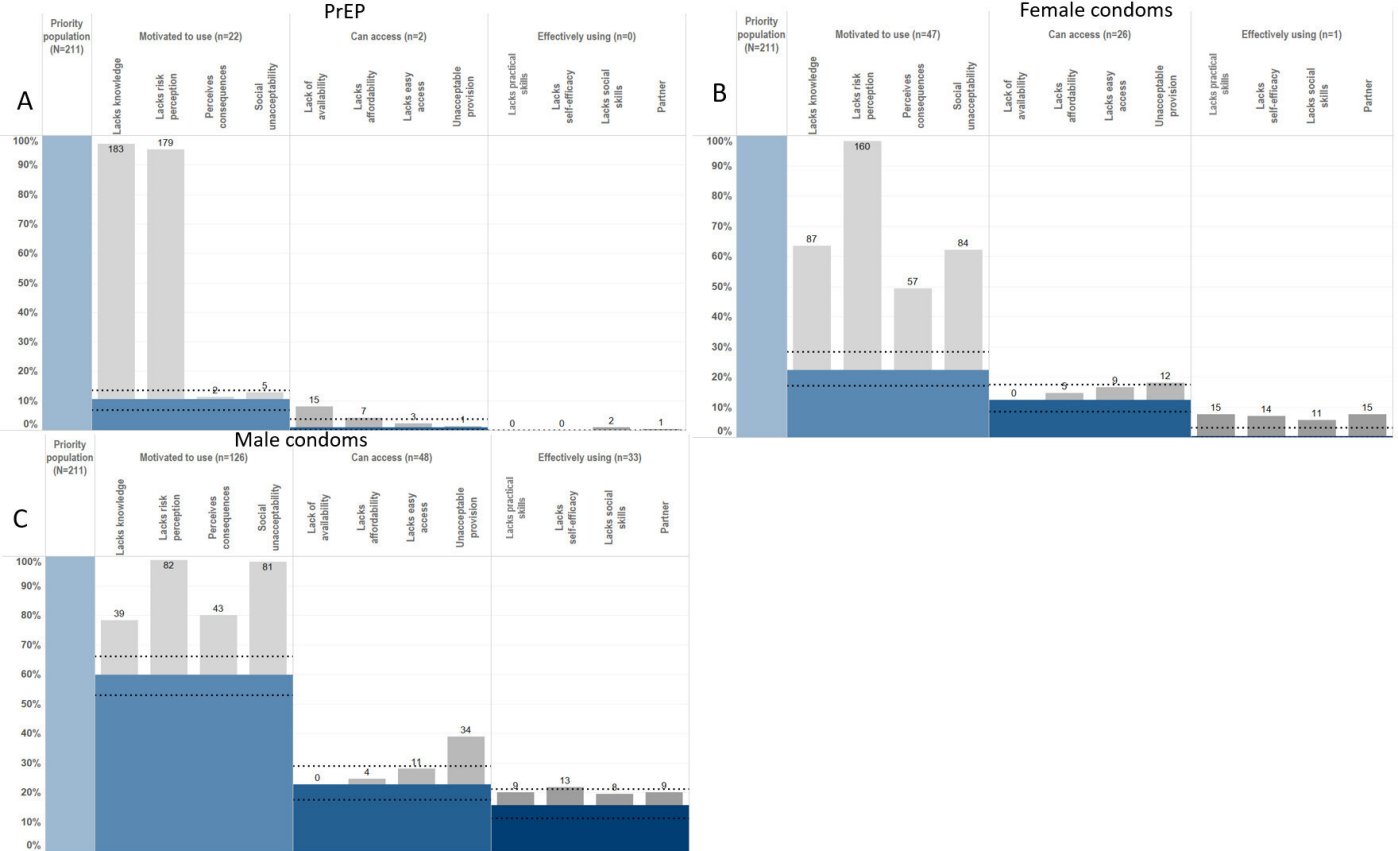
HIV prevention cascades for (A) pre-exposure prophylaxis (PrEP), (B) female condom and (C) male condom use in young women.

Individual HIV-HPCs—for PrEP, female condoms and male condoms—were populated for HIV-negative AGYW reporting ≥1 risk behaviour. Among these AGYW, 10% (22/211) reported being motivated to use PrEP (figure 1A). Lack of knowledge of PrEP (97%) was the largest barrier in women not reporting motivation. A high percentage (95%) of women not motivated also did not perceive a future risk of HIV infection. Of the AGYW who were motivated, 91% (20/22) could not access PrEP, with 75% of these reporting lack of availability. No women in the priority population reported PrEP use.

Motivation among AGYW to use female condoms was low (figure 1B). Only 22% (47/211) reported motivation and <1% reported using female condoms as an HIV prevention method. Lack of risk perception, followed by lack of knowledge and social unacceptability, was the biggest barrier in the cascade.

For male condoms, 60% of AGYW were motivated, 23% had access and 16% were using the method (figure 1C). Lack of risk perception (96%) was the most common barrier to motivation; however, social unacceptability was also commonly reported (95%). Sixty-two per cent of motivated AGYW reported lacking access to male condoms, and 44% of this reported lacking acceptable provision (embarrassment or lack of privacy/confidentiality). Thirty-one per cent of AGYW who were motivated and had access to male condoms were not effectively using them. Lack of self-efficacy (defined here as an individual’s confidence in their ability to perform specific actions, such as negotiating or using condoms effectively including lacking capacity to use condoms due to family or peer disapproval) was the biggest barrier to effective use (87% of those with motivation and access but not using condoms). 60% of those with motivation and access but not effective use reported lacking partner acceptance to use male condoms.

### HIV prevention cascades for ABYM in the priority population (reporting sexual risk behaviours)

**Figure 2:**
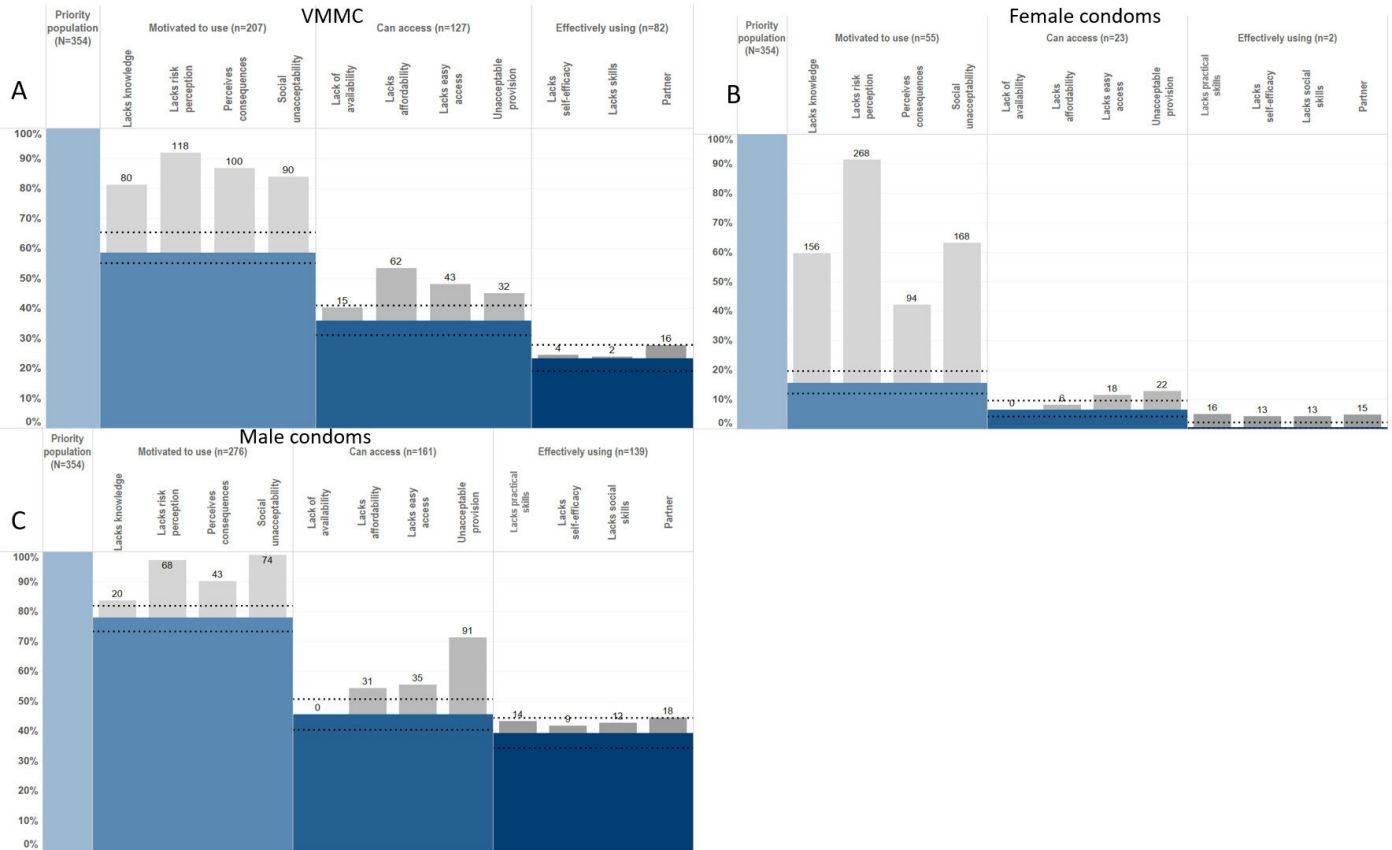
HIV prevention cascades for (A) voluntary medical male circumcision (VMMC), (B) female condom and (C) male condom use in young men.

Individual HIV-HPCs—for VMMC, female condoms and male condoms—were populated for HIV-negative ABYM reporting ≥1 risk behaviour. Among the priority population of HIV-negative ABYM reporting ≥1 risk behaviour, 58% were motivated to use VMMC, 36% could access it and 23% were fully circumcised (figure 2A). Fifty-four per cent of unmotivated ABYM lacked knowledge of VMMC as an HIV prevention method, 80% did not perceive HIV risk and 68% perceived negative consequences (painful procedure, irreversible procedure, loss of sexual pleasure). Thirty-nine per cent of ABYM motivated to use VMMC could not access it, and 78% of these ABYM reported affordability as a barrier. Of the ABYM who were motivated and had access, 35% were not circumcised and their largest barrier was lack of partner acceptance (36%).

Seventy-eight per cent of ABYM were motivated, 45% had access to and 39% were using male condoms (figure 2C). Ninety-two per cent of ABYM lacking motivation lacked perception of future risk of HIV infection, 55% perceived negative consequences of condom use (reporting reduced sexual pleasure) and 95% reported lack of social acceptability as a barrier to motivation. The drop between motivation and access was the largest drop in this cascade, with 42% of motivated individuals reporting lack of access. Seventy-nine per cent of the men who lacked access reported unacceptable provision of male condoms and 27% reported cost as a barrier. Fourteen per cent of ABYM who were motivated to use and had access to male condoms were not using them effectively, with 82% of these reporting lack of partner acceptance. Lack of self-efficacy to use male condoms was a larger barrier in AGYW than in the equivalent ABYM group: 87% of AGYW vs 30% of ABYM (OR 9.39; 95% CI 1.69 to 52.13). Reported use of male condoms was higher in men than in women (OR 1.15; 95% CI 0.59 to 2.28), and higher than for PrEP or VMMC; however, no statistically significant differences were observed. Use of female condoms was low: 16% of men reported motivation to use them, and <1% reported actual use (figure 2B).

### Combination prevention method use in the AGYW and ABYM priority populations

**Figure 3:**
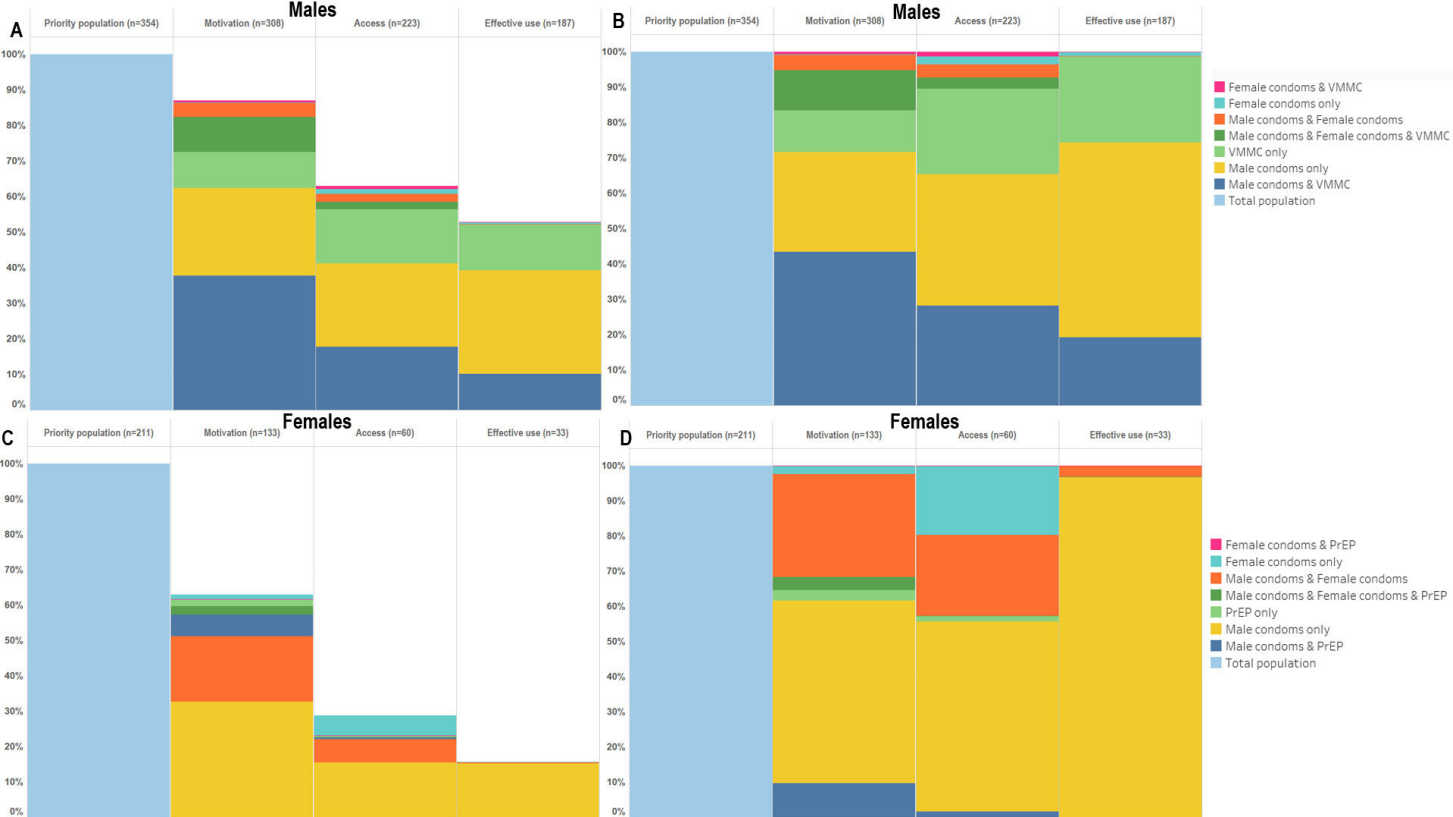
HIV combination prevention cascades showing overall male (A & B) and female (C & D) prevention gaps and barriers to use and breakdowns by prevention method. PrEP, pre-exposure prophylaxis; VMMC, voluntary medical male circumcision.

Overall, 63% of AGYW in the priority population were motivated, 28% had access to and 16% reported to be effectively using ≥1 HIV prevention method (figure 3A). Use of male condoms accounted for 97% of prevention method use (figure 3B). Of ABYM in the priority population, 87% were motivated to use, 63% had access to and 53% were using ≥1 method (figure 3C). Use of male condoms accounted for 55% of prevention method use, followed by VMMC alone (25%) (figure 3D).

## Discussion

This analysis presents the first instance of the Harare HIV-CPC framework being fully populated with general population survey data. Using the Harare HIV-CPC framework, we measured combination and individual primary prevention method use among young people at risk of acquiring HIV, and quantified their particular barriers to individual prevention method use. Barriers were present at all steps of the cascade, indicating that multilevel determinants of prevention method use will need to be targeted by interventions to reduce HIV incidence in young people.

Reported HIV combination prevention cascades were presented in this analysis—one of the first instances of HPCs being applied this way. Levels of motivation, access to and effective use of any prevention method were markedly lower in AGYW than in ABYM, with only 16% of AGYW reporting use of any prevention method compared with 53% of ABYM. Male condoms remain the most popular (ie, the method for which people report motivation), accessible and most widely used primary prevention method, accounting for almost all reported prevention method use. The gap between motivation and access is the largest gap in the combination cascade. Motivation to use ≥1 prevention method is particularly high in ABYM: 87% of ABYM in the priority population reported wanting to use at least one prevention method. The UNAIDS 2025 Roadmap includes goals of linking at least 90% of people at heightened risk of HIV infection to services, prioritising HIV prevention packages and ensuring they are used by 95% of those at risk of HIV infection.^17^ We found that 37% of ABYM and 19% of AGYW who have started sex reported ≥1 HIV risk behaviour in the last 12 months, despite declines in risk behaviours observed in earlier studies in Manicaland,^2^ although trends in eastern and southern Africa vary.^26^ The proportions of the priority populations reporting motivation and access to ≥1 prevention method in this study fall below these targets. Levels of use of prevention methods also remain below targets set out in Zimbabwe’s National HIV and AIDS Strategic Plan,^27^ even when assessing use of prevention methods in combination. Estimates of prevention method use presented here are lower than some other published estimates of condom use specifically at last sex with a non-regular partner, such as in the recent Zimbabwean Demographic and Health Survey.^28^ This is likely due to the priority population being more broadly defined and likely includes individuals in long-term relationships where condom use tends to be lower.

Motivation, access and effective use were consistently lower in AGYW than in ABYM for all prevention methods—a key issue given the excess HIV incidence observed in AGYW.^11^ This contradicts other reports of generally lower engagement and retention in HIV treatment, testing and prevention services among men in eastern and southern Africa, although estimates vary depending on the population, definition of high-risk sexual behaviour within the denominator of estimates and definition and context of prevention method use.^29^ The reliance on self-reported data may mean estimates of prevention method use were higher due to social desirability biases influencing reporting. Differences in reporting biases of both sexual risk behaviour and prevention method use between men and women are likely to be present.^30 31^ Inclusion of VMMC—a one-off procedure—within the male measure of combination prevention may increase the relative estimate of prevention use in men compared with women. The recent availability within PrEP in the study area—and observed lack of knowledge about PrEP—means that reported PrEP use among AGYW is lower than in other study areas with wider PrEP availability. The Harare HIV-CPC framework provides insight into individual motivation to use prevention methods. The notable number of individuals lost from the HIV-CPC between the motivation and effective use bars in both young men and women suggests that even where there is demand for primary HIV prevention, other barriers prevent motivated individuals from actually using it. Motivation to use, access to and use of female condoms were low compared with male condoms, but comparable with other estimates of female condom use within the region, which range from 3% to 38%.^32^ Motivation to use, access to and effective use of PrEP were particularly low. Lack of knowledge of PrEP was the biggest barrier to motivation to use, which, together with the reports of poor availability and affordability, reflects the recent introduction of PrEP in Manicaland at the time. Qualitative research carried out in the same population also identified an overall lack of awareness of where to access PrEP.^33^ Concerns about disclosure of PrEP use to the partner and struggles to take PrEP discretely and consistently were identified from this work,^33^ although these were not observed in the HPC due to most people being lost from the cascade at earlier steps.

Both AGYW and ABYM reported highest motivation for, access to and actual uptake of male condoms among all prevention methods analysed, reflecting the history of male condom programmes and availability within Zimbabwe and the wider region of southern Africa. Despite this, the gap between motivation and access was sizeable. A lack of self-efficacy—reporting lack of confidence to use male condoms regularly or to use them despite partner, peer or family disapproval—was indicated as a barrier by the HPC, and has been found to be associated with lower odds of condom use in both men and women.^21^ There was a large drop-off between the motivation and access to male condoms for both ABYM and AGYW, highlighting that access-related barriers need to be addressed.

VMMC levels reported are well below the target of 80% of men aged 15–29 years set out in Zimbabwe’s National HIV and AIDS Strategic Plan, with only 23% of ABYM reporting full medical male circumcision.^27^ Perceived negative consequences of VMMC were reported by 68% of ABYM unmotivated to have VMMC. Lack of affordability—likely related to time off work for the procedure and recovery—was the most commonly reported barrier to accessing VMMC. However, lack of easy access and lack of acceptable provision were also commonly reported, with more than half of those who had motivation but not access reporting a lack of easy access to VMMC services.

Lack of self-perceived future risk of HIV was a common barrier across prevention methods and has been shown to be associated with high HIV incidence.^34^ Lack of accurate risk perception has been noted in young people in our study population in east Zimbabwe.^34^ A lack of social acceptability was a commonly reported barrier across multiple prevention methods (male condoms, female condoms, VMMC) by both ABYM and AGYW. Partner resistance was a commonly reported barrier by AGYW for use of both female and male condoms.

Use of self-reported data could cause underestimation of the priority population (despite use of secret voting methods^22^) and overestimation of effective use of prevention methods, as also noted by Kelly *et al* in a recent Zimbabwean population survey.^35^ These analyses only assess a cross-section of risk behaviour and prevention method use, relying on the assumption that these remain the same in the future—a particular issue for young people whose sexual behaviour can change over short periods of time.^36^ Analyses do not explore differences in the HPCs according to the type of self-reported risk behaviour. Only the main bars of the HIV-CPC were presented due to the complexity of populating explanatory barriers for multiple prevention methods in one cascade.

Despite these limitations, our findings demonstrate the value and utility of measuring the Harare HIV-CPC framework in a population survey. For AGYW and ABYM, they highlight the need for interventions in all parts of the cascades. Furthermore, the variations found between the gaps and barriers for different HIV prevention methods and priority populations point to a need for interventions, which are specific to particular methods and populations as well as the local context. Nevertheless, some barriers are common across prevention methods and could be targeted by broader, cross-cutting interventions. For example, appropriate community-level interventions could improve knowledge of risky sexual behaviours and reduce social unacceptability by addressing the stigma attached to use of prevention methods. Interventions which increase the privacy and confidentiality of prevention method providers, and public confidence in this privacy, could reduce unacceptable provision as a barrier. The role of partner as a barrier in the capacity to use prevention methods was consistently reported across multiple methods. Interventions to improve AGYW’s capacity to negotiate prevention method use with a partner and acceptance of use within a partnership could increase prevention method use.

## Conclusions

The HIV-CPC has enabled identification of barriers to motivation (particularly knowledge of PrEP, social acceptability of condoms and VMMC and current and future risk perception), access (particularly availability of PrEP, and acceptable provision of all primary prevention methods) and, ultimately, effective use of primary HIV prevention methods (particularly the practical and social skills required to negotiate use of primary prevention methods with a sexual partner). These barriers vary by priority population and prevention method and could be targeted by interventions to improve effective use, including increasing motivation to use prevention methods and removing fear of stigma and judgement of prevention method use in ABYM and AGYW. High proportions of young people engaging in risky sexual behaviour remain, indicating a need to improve HIV prevention method use to prevent acquisition of HIV. Even when young people are motivated and have access to prevention, barriers remain including lack of social skills and self-efficacy to negotiate prevention method use.

## Supplementary material

10.1136/bmjph-2025-002860 online supplemental file 1

10.1136/bmjph-2025-002860 online supplemental file 2

## Data Availability

Data are available on reasonable request.
